# T2^*∗*^ Relaxation Time Obtained from Magnetic Resonance Imaging of the Liver Is a Useful Parameter for Use in the Construction of a Murine Model of Iron Overload

**DOI:** 10.1155/2019/7463047

**Published:** 2019-09-22

**Authors:** Yukari Matsuo-Tezuka, Yusuke Sasaki, Toshiki Iwai, Mitsue Kurasawa, Keigo Yorozu, Yoshihito Tashiro, Michinori Hirata

**Affiliations:** Product Research Department, Chugai Pharmaceutical Co. Ltd., Kamakura, Japan

## Abstract

**Aim:**

Iron overload is a life-threatening disorder that can increase the risks of cancer, cardiovascular disease, and liver cirrhosis. There is also a risk of iron overload in patients with chronic kidney disease. In patients with renal failure, iron storage is increased due to inadequate iron utilization associated with decreased erythropoiesis and also to the inflammatory status. To evade the risk of iron overload, an accurate and versatile indicator of body iron storage in patients with iron overload is needed. In this study, we aimed to find useful iron-related parameters that could accurately reflect body iron storage in mice in order to construct a murine model of iron overload.

**Methods:**

To select an appropriate indicator of body iron status, a variety of parameters involved in iron metabolism were evaluated. Noninvasively measured parameters were R1, R2, and R2^*∗*^ derived from magnetic resonance imaging (MRI). Invasively measured parameters included serum hepcidin levels, serum ferritin levels, and liver iron contents. Histopathological analysis was also conducted.

**Results/Conclusion:**

Among the several parameters evaluated, the MRI T2^*∗*^ relaxation time was able to detect iron storage in the liver as sensitively as serum ferritin levels. Moreover, it is expected that using an MRI parameter will allow accurate evaluation of body iron storage in mice over time.

## 1. Introduction

Iron is an essential element for biological function. However, excess iron has the potential for life-threatening consequences via production of reactive oxygen species (ROS). It is reported that under iron-overload conditions, mitochondrial DNA and organs are damaged by ROS produced by the labile iron pool [1]. A typical iron-overload disease is hereditary hemochromatosis. It is characterized by dysregulated iron absorption and subsequent overaccumulation of iron in various tissues such as the liver, pancreas, heart, and joints [2]. Prolonged iron-overload conditions cause liver cirrhosis, liver cancer, and diabetes [3, 4].

To evade iron-overload disease, it is crucial to assess body iron storage in an appropriate manner. However, it is difficult to know the exact status of body iron storage because iron metabolism is influenced by various factors such as inflammation, erythropoiesis, and disease conditions [5]. A typical example of dysregulated iron metabolism is found in chronic kidney disease (CKD) [6]. In patients with chronic renal failure, damaged kidneys produce less erythropoietin than do healthy kidneys, which causes a reduction in erythropoietic activity and consequently a reduction in iron utilization for hemoglobin synthesis. As a result, iron storage is increased in CKD patients. Moreover, in patients with CKD, production of proinflammatory cytokines is also increased in association with renal failure and uremia. Cytokines such as IL-6 are known to dysregulate iron homeostasis via the upregulation of hepcidin, a key regulator of iron homeostasis [7]. Thus, it is difficult to accurately assess body iron status in patients with inflammation, erythropoietic failure, and dysregulated iron homeostasis. Considering the risk posed by dysregulation of iron homeostasis, especially the risk of iron overload, development of an accurate and versatile indicator of body iron storage is necessary.

As indicators of body iron storage, serum ferritin level is usually used in the clinical setting as is the concentration of serum/plasma hepcidin, which is reported to correlate well with liver iron concentration [8, 9]. Although these are commonly used, there are limits to their application because serum ferritin and hepcidin levels are easily influenced by inflammatory status and erythropoietic status. Although liver biopsy is infrequent in the clinical setting, its versatility is low in terms of its invasiveness. Magnetic resonance imaging (MRI) is coming to be used in clinical settings as a tool with which to assess body iron storage, not only visually but also quantitatively [10]. One of the advantages of MRI is its noninvasiveness, allowing MRI to be used for sequential assessment of target atoms/molecules. MRI is also considered to be advantageous for assessment of body iron status in that it is not influenced by inflammation-mediated disorders of iron metabolism. However, the use of MRI is not popular due to its high cost and poor access. In summary, although there are various parameters used for assessing body iron status in the clinical setting, each has its advantages and disadvantages. In this study, we sought an optimal indicator of body iron storage for use in nonclinical research.

In nonclinical research with mice, liver iron concentration can be used as an absolute reference for body iron storage. We constructed an iron loading model by injecting mice with different dosages of iron-dextran and measured various iron parameters to select effective biomarkers for body iron storage. As invasive indicators of body iron storage, we measured serum ferritin and hepcidin and evaluated their correlations with liver iron concentration. As noninvasive indicators, T1, T2, and T2^*∗*^ relaxation time of the liver were measured by an MRI system designed for use with mice. We evaluated the correlations between liver iron concentration and other iron-related parameters including MRI parameters.

## 2. Materials and Methods

### 2.1. Chemicals and Antibodies

Iron-dextran and Dulbecco's phosphate-buffered saline were purchased from Sigma-Aldrich (St. Louis, MO, USA). All other chemicals and solvents were of analytical reagent grade.

### 2.2. Animals

Seven-week-old male C57BL/6NCrlCrlj mice were purchased from Charles River Laboratories Japan (Kanagawa, Japan). All animals were allowed to acclimatize for 8 days to recover from shipping-related stress prior to the study. Mice were housed under specific pathogen-free conditions with free access to food and water. All studies were approved by the Institutional Animal Care and Use Committee at Chugai Pharmaceutical Co., Ltd., and conformed to the Institute for Laboratory Animal Research (ILAR).

### 2.3. Animal Treatment

Iron-dextran was diluted to appropriate concentrations in phosphate buffer vehicle (phosphate-buffered saline containing 0.02% polyoxyethylene sorbitan monooleate (Tween 80)). Iron-loaded mice were prepared by intraperitoneal injection of iron-dextran (0.1, 0.5, or 2.5 mg/mouse). Three days after iron or vehicle loading, MRI of mice was taken, followed by euthanization by exsanguination under anesthesia with isoflurane. Five mice from each group were used.

### 2.4. Magnetic Resonance Imaging of Hepatic Iron Stores

Mice were anesthetized by exposure to isoflurane and maintained under anesthesia during the experiment. Magnetic resonance images were acquired using an MRI system (Agilent, Santa Clara, CA, USA) for collecting and analyzing parametric maps of the mouse liver at 7T (300 MHz). Breathing rate was monitored by the scanner with a pressure sensitive respiratory monitor (SA Instruments, Stony Brook, NY). To avoid respiratory-related motion artifacts, isoflurane levels were modulated as necessary to maintain the respiratory rate at 30 ± 10 breaths per minute. Proton-density-weighted images were acquired using a fast spin-echo multislice sequence (TR = 1500 ms, TE = 10 ms, echo train length = 8, kzero = 1, 128 × 128 matrix, field-of-view (FOV) = 40 × 40 mm, thickness = 1 mm). For the quantification of T1 values, we used an inversion recovery gradient echo multislice sequence which allowed the acquisition of a single T1 map (TR = 4.64 ms, TE = 1.96 ms, inversion recovery time = 0.1, 1.075, 2.05, 3.025, 4.0 s, flip angle 10°, 64 × 64 matrix, FOV = 40 × 40 mm, thickness = 2 mm). T2 values were obtained using a fast spin-echo multislice sequence (TR = 1500 ms, TE = 8, 11, 16, 22 ms, echo spacing = 8.35 ms, kzero = 1, 128 × 128 matrix, FOV = 40 × 40 mm, thickness = 2 mm). T2^*∗*^ values were obtained using gradient echo multislice sequence (TR = 100 ms, TE = 3, 5, 8, 12 ms, flip angle 20°, 128 × 128 matrix, FOV = 40 × 40 mm, thickness = 2 mm). Images were analyzed using VnmrJ software (Agilent) whereby T1 and T2, T2^*∗*^ parameter maps were calculated from acquired images. The relaxation rate was calculated as follows: R1 = 1/T1, R2 = 1/T2, and R2^*∗*^=1/T2^*∗*^.

### 2.5. Specimen Collection

Blood was collected and divided into two aliquots. The first aliquot was collected into Minicollect ethylenediaminetetraacetic acid tubes (Greiner Bio-One, Kremsmünster, Austria). The second aliquot was collected into evacuated blood-collecting tubes (Terumo Corporation, Tokyo, Japan), and serum was isolated according to the manufacturer's instructions. Part of the liver was harvested for histological analysis and fixed in 10% neutral buffered formalin. The remaining part of the liver was used for iron content analysis.

### 2.6. Measurement of Hematological and Iron Indices in the Blood

Hematological indices were measured by an automated hematology analyzer (XT-2000iV; Sysmex, Hyogo, Japan). Serum iron levels as well as unsaturated iron binding capacity (UIBC) and total iron binding capacity (TIBC) were measured using an automatic biochemistry analyzer (TBA-120FR, Toshiba Medical Systems, Tochigi, Japan). Serum hepcidin level was measured by liquid chromatography/electrospray ionization tandem mass spectrometry using an AB Sciex Triple Quad 5500 system (AB Sciex, Foster City, CA, USA) equipped with a Prominence UFLCXR system (Shimadzu Corporation, Kyoto, Japan) as reported previously [11]. Serum ferritin levels were determined by ELISA kit (ALPCO, Salem, NH, USA).

### 2.7. Measurement of Iron Content in Liver

Liver samples were first dissolved in nitric acid and decomposed by heating. Iron concentrations were then measured by inductively coupled plasma-atomic emission spectrometry (ICP-AES, Yagai-Kagaku Co., Sapporo, Japan).

### 2.8. Histopathology

Sections (4 *μ*m thick) were prepared from paraffin-embedded formalin-fixed liver samples. Liver hemosiderin deposition was assessed by Berlin blue staining.

### 2.9. Statistical Analysis

All values are shown as mean and standard deviation (SD). Statistical analysis was performed using JMP version 11.2.1 software (SAS Institute, Cary, NC, USA). Comparisons between groups were assessed by Dunnett's test. A *P* value of less than 0.05 was used to estimate statistical significance. Linear approximation and correlation coefficients were also analyzed.

## 3. Results

### 3.1. Noninvasive MRI Analysis of Liver Iron Storage

**Figure 1:**
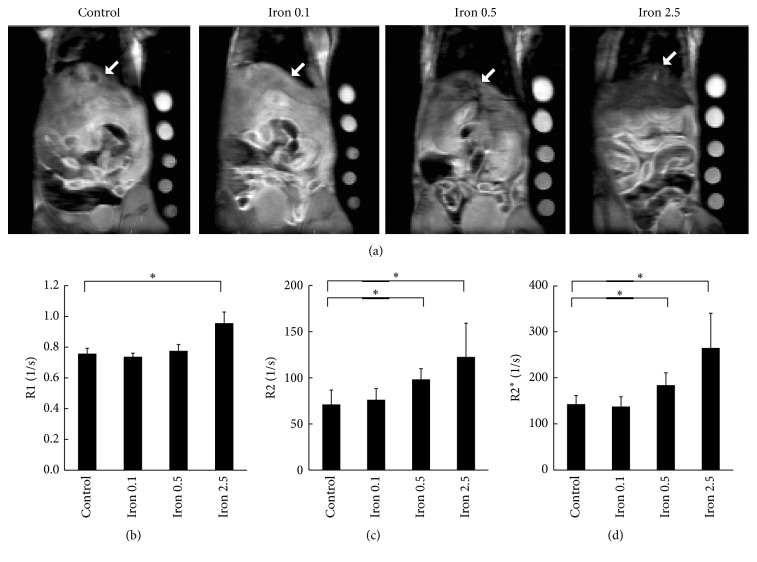
(a) Representative proton-density-weighted images of livers (indicated by white arrow) on Day 3 after administration of iron-dextran or dextran (control = dextran; Iron 0.1 = 0.1 mg iron-dextran/mouse; Iron 0.5 = 0.5 mg iron-dextran/mouse; Iron 2.5 = 2.5 mg iron-dextran/mouse). (b) R1, (c) R2, and (d)R2^*∗*^ of MRI of livers on Day 3 after administration of iron-dextran or dextran (control = dextran; Iron 0.1 = 0.1 mg iron-dextran/mouse; Iron 0.5 = 0.5 mg iron-dextran/mouse; Iron 2.5 = 2.5 mg iron-dextran/mouse). Results are expressed as mean + SD. Five mice from each group were used. Statistical significances were analyzed by Dunnett's test. ^*∗*^*P* < 0.05.

We used MRI to assess liver iron storage in 4 groups with different body iron status: the control group and the 0.1, 0.5, and 2.5 mg iron-dextran loaded groups. Representative proton-density-weighted images are shown in Figure 1(a). Mean R1, R2, and R2^*∗*^ are shown in Figures 1(b)–1(d), respectively. The R1 could detect the difference between the control group and the group loaded with 2.5 mg of iron-dextran. The R2 and R2^*∗*^ could detect the difference between the control group and the group loaded with 0.5 mg of iron-dextran as well as the difference between the control group and the group loaded with 2.5 mg of iron-dextran.

### 3.2. Invasive Analysis of Iron Parameters in Serum and Liver Samples

**Figure 2:**
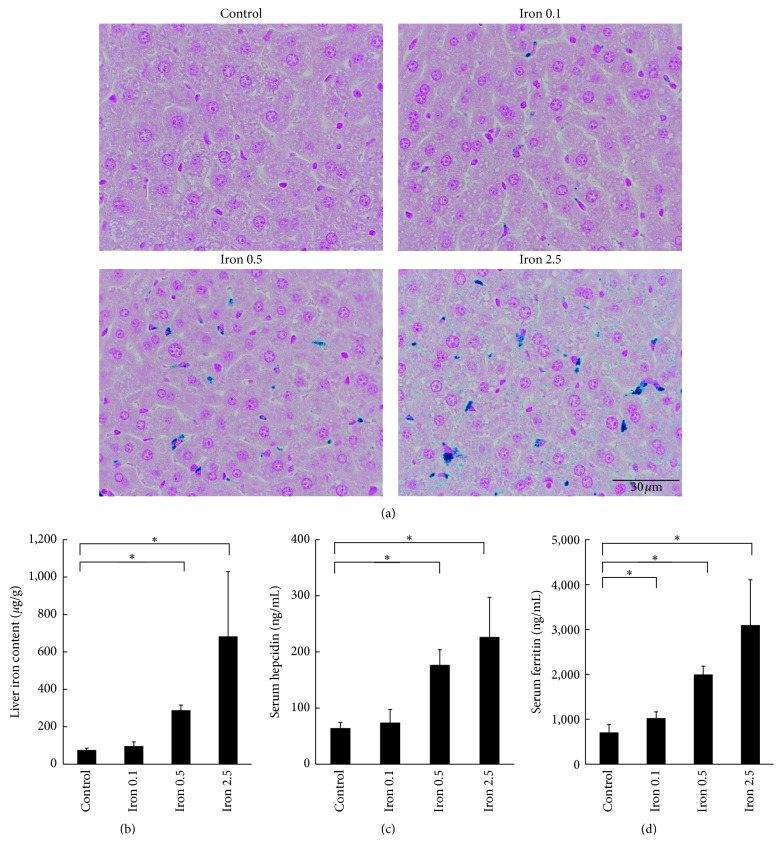
(a) Histopathological analysis for hepatic hemosiderin deposition in iron-loaded mice on Day 3 after administration of iron-dextran or dextran (control = dextran; Iron 0.1 = 0.1 mg iron-dextran/mouse; Iron 0.5 = 0.5 mg iron-dextran/mouse; Iron 2.5 = 2.5 mg iron-dextran/mouse). (b) Liver iron content, (c) serum hepcidin levels, and (d) serum ferritin levels on Day 3 after administration of iron-dextran or dextran (control = dextran; Iron 0.1 = 0.1 mg iron-dextran/mouse; Iron 0.5 = 0.5 mg iron-dextran/mouse; Iron 2.5 = 2.5 mg iron-dextran/mouse). Results are expressed as mean + SD. Five mice from each group were used. Statistical significances were analyzed by Dunnett's test. ^*∗*^*P* < 0.05.

Specimens were collected to evaluate a variety of iron parameters such as hemosiderin deposition in the liver, liver iron content, serum hepcidin levels, and serum ferritin levels. Representative images of hemosiderin deposition visualized by Berlin blue staining are shown in Figure 2(a). As were shown by the R1, R2, and R2^*∗*^ obtained from MRI, hemosiderin deposition tended to increase with iron loading dose. Liver iron content (Figure 2(b)) and serum hepcidin levels (Figure 2(c)) could detect the difference between the 0.5 mg iron-dextran loading group and the control group. Serum ferritin levels (Figure 2(d)) could detect the difference between the 0.1 mg iron-dextran loading group and the control group. On the other hand, hematological parameters and serum iron concentration were not increased by these levels of iron loading (Table 1).

### 3.3. R2^*∗*^ and Serum Ferritin Level Were Correlated Most Strongly with Liver Iron Content among All Iron-Related Parameters

**Figure 3:**
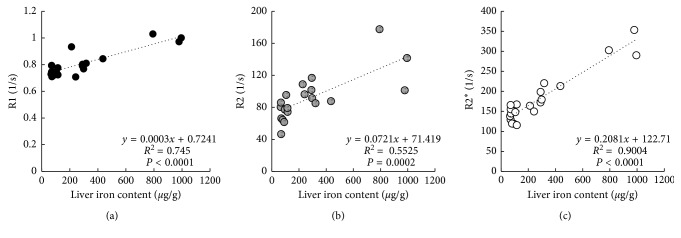
Correlations of (a) R1 value obtained from MRI of liver *vs.* liver iron content, (b) R2 value obtained from MRI of liver *vs.* liver iron content, and (c) R2^*∗*^ value obtained from MRI of liver *vs.* liver iron content.

**Figure 4:**
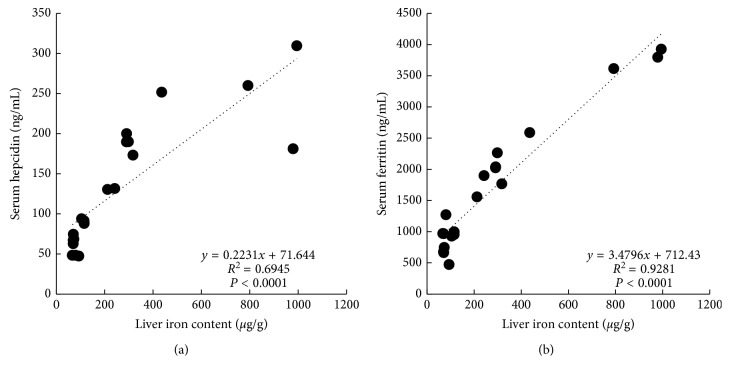
Correlations of (a) serum hepcidin level *vs.* liver iron content and (b) serum ferritin level vs. liver iron content.

To investigate how well each indicator reflected body iron storage in each iron-loading model, correlation analyses between liver iron content and all other iron-related parameters were conducted. Analysis of the R1, R2, and R2^*∗*^ (Figures 3(a)–3(c)) revealed that the R2^*∗*^ was the MRI parameter that correlated most highly with liver iron content (*R*^2^ = 0.9004). Among the invasive iron-related parameters (Figures 4(a) and 4(b)), serum ferritin levels were most strongly correlated with liver iron content (*R*^2^ = 0.9281). According to these comparison studies, as a noninvasive parameter, the T2^*∗*^ relaxation time obtained from MRI of the liver is an effective indicator of body iron status, and as an invasive parameter, serum ferritin level is also an effective indicator of body iron status.

## 4. Discussion

As shown in iron-overload disorders such as hemochromatosis and *β*-thalassemia, excess iron is detrimental to the body [4, 12]. To monitor body iron status in people with these conditions, serum ferritin and hepcidin are generally used as biomarkers for iron storage. However, it is difficult to assess body iron storage in patients with inflammation or infection because several inflammatory cytokines are produced under such conditions and iron-related parameters such as serum ferritin and hepcidin are influenced by such factors. A typical example of dysregulated iron metabolism is CKD. In patients with renal failure, damaged kidneys cannot produce adequate erythropoietin, a humoral factor promoting proliferation and maturation of erythroid cells, and consequently hemoglobin synthesis is downregulated. This leads to an increase in stored iron. In some patients with CKD, iron metabolism is further complicated because of inflammatory status [13, 14]. To make matters worse, one of the therapeutic approaches for renal anemia is iron supplementation. To evade the risk of excess iron accumulation, it is crucial to make use of an iron storage indicator that reflects body iron storage simply and accurately. An appropriate marker for body iron storage is necessary not only for patients with CKD but for all patients with dysregulated iron metabolism to evade the risk of tissue damage evoked by excess iron.

To select an appropriate indicator of body iron storage, we evaluated noninvasively and invasively measured iron-related parameters in mice loaded with different dosages of iron-dextran. Among the parameters evaluated, the MRI-derived T2^*∗*^ relaxation time as a noninvasive parameter and serum ferritin level as an invasive parameter were shown to be relatively accurate indicators of body iron storage. Moreover, serum ferritin level was the most sensitive indicator of body iron storage because this was the only parameter that could detect the difference between the control group and the group administered 0.1 mg iron-dextran/mouse. Although serum ferritin level is an accurate and sensitive indicator of body iron storage, this parameter is known to be influenced by inflammatory status [15]. Moreover, even under normal conditions, mouse serum ferritin levels are quite high compared with human levels. Therefore, when considering application to humans, serum ferritin level is not always optimal as an index of body iron storage. Among circulating iron-related parameters, serum iron was not associated with the dosage of iron-dextran (Table 1), and therefore it was considered to be inappropriate as an index of iron storage. On the other hand, the MRI-derived T2^*∗*^ relaxation time is not only a sensitive indicator of body iron storage but is also a parameter that can be measured noninvasively. Taking into account its accuracy and its capability to assess changes in body iron storage over time, we concluded that the MRI-derived T2^*∗*^ relaxation time is the most appropriate indicator for assessing body iron storage. This parameter is known to be appropriate for measuring the paramagnetic effect of iron in the clinical setting [16], which supports the superiority of T2^*∗*^ relaxation time in assessing liver iron concentration in a murine model of iron overload.

The spleen is another iron storage organ. We evaluated T2^*∗*^ relaxation time of the spleen as well as the liver and analyzed its correlation with spleen iron content. However, the T2^*∗*^ relaxation time could not accurately evaluate spleen iron content because the tissue composition of the spleen was incompatible with MRI measurement (data not shown).

Among the several types of iron overload model, iron loading by administration of iron-dextran can be regarded as a model for secondary hemochromatosis. Because parenteral iron supplementation is the simplest and easiest way to achieve predetermined iron storage levels, research on secondary hemochromatosis is expected to be accelerated even without the use of genetically modified mice. Moreover, by using the T2^*∗*^ relaxation time obtained from MRI, it is possible to assess body iron status in the model prior to the study and during the study without sacrifice. In the clinical setting, the association between iron storage and tissue damage has not been adequately elucidated yet. The combination of a murine parenteral iron loading model and iron assessment by MRI could help us study the pathology of dysregulated iron metabolism.

In summary, it is shown that MRI designed for small animals is helpful for assessing body iron status in mice, and from among several iron-related parameters, the T2^*∗*^ relaxation time obtained from MRI could detect iron storage sensitively and accurately. Therefore, it is possible to conduct research on iron metabolism in mice to a greater depth than ever before. However, it remains a challenge to take the findings from research on iron metabolism in mice and apply them in the clinical setting. To confirm that iron-related indices in mice are applicable to humans, invasive and noninvasive indices associated with iron metabolism need to be investigated in both mice and humans.

## Figures and Tables

**Table 1:** Hemoglobin levels and iron indices in the blood on Day 3 after administration of iron-dextran or dextran (control = dextran group; Iron 0.1 = 0.1 mg iron-dextran/mouse; Iron 0.5 = 0.5 mg iron-dextran/mouse; Iron 2.5 = 2.5 mg iron-dextran/mouse).

	Control	Iron 0.1	Iron 0.5	Iron 2.5
Hemoglobin (g/dL)	15.0 ± 1.1	15.3 ± 1.6	15.8 ± 1.5	14.3 ± 0.6
Serum iron (*μ*g/dL)	152.8 ± 42.7	182.8 ± 46.8	143.6 ± 27.2	184.6 ± 22.0
UIBC (*μ*g/dL)	120.0 ± 13.0	121.8 ± 22.2	140.8 ± 24.3	130.6 ± 30.4
TIBC (*μ*g/dL)	272.8 ± 35.7	304.6 ± 45.8	284.4 ± 26.9	315.2 ± 27.2

Mean ± SD (*n* = 5); UIBC, unsaturated iron binding capacity; TIBC, total iron binding capacity.

## Data Availability

The data used to support the findings of this study were provided by Chugai Pharmaceutical Co., Ltd., under license and so cannot be made freely available. Access to these data will be considered by the corresponding author upon request, with the permission of Chugai Pharmaceutical Co., Ltd.

## References

[1] Dixon S. J., Stockwell B. R. (2014). The role of iron and reactive oxygen species in cell death. *Nature Chemical Biology*.

[2] Worthen C. A., Enns C. A. (2014). The role of hepatic transferrin receptor 2 in the regulation of iron homeostasis in the body. *Frontiers in Pharmacology*.

[3] Lagergren K., Wahlin K., Mattsson F., Alderson D., Lagergren J. (2016). Haemochromatosis and gastrointestinal cancer. *International Journal of Cancer*.

[4] Powell L. W., Seckington R. C., Deugnier Y. (2016). Haemochromatosis. *The Lancet*.

[5] Ganz T., Nemeth E. (2015). Iron homeostasis in host defence and inflammation. *Nature Reviews Immunology*.

[6] Zumbrennen-Bullough K., Babitt J. L. (2014). The iron cycle in chronic kidney disease (CKD): from genetics and experimental models to CKD patients. *Nephrology, Dialysis, Transplantation*.

[7] Nemeth E., Rivera S., Gabayan V. (2004). IL-6 mediates hypoferremia of inflammation by inducing the synthesis of the iron regulatory hormone hepcidin. *The Journal of Clinical Investigation*.

[8] Kanwar P., Kowdley K. V. (2013). Diagnosis and treatment of hereditary hemochromatosis: an update. *Expert Review of Gastroenterology & Hepatology*.

[9] Wish J. B. (2006). Assessing iron status: beyond serum ferritin and transferrin saturation. *Clinical Journal of the American Society of Nephrology: CJASN*.

[10] Anderson L. J. (2011). Assessment of iron overload with T2^*∗*^ magnetic resonance imaging. *Progress in Cardiovascular Diseases*.

[11] Murao N., Ishigai M., Yasuno H., Shimonaka Y., Aso Y. (2007). Simple and sensitive quantification of bioactive peptides in biological matrices using liquid chromatography/selected reaction monitoring mass spectrometry coupled with trichloroacetic acid clean-up. *Rapid Communications in Mass Spectrometry: RCM*.

[12] Liaska A., Petrou P., Georgakopoulos C. D. (2016). beta-Thalassemia and ocular implications: a systematic review. *BMC Ophthalmology*.

[13] Antunes S. A., Canziani M. E. (2016). Hepcidin: an important iron metabolism regulator in chronic kidney disease. *Jornal Brasileiro de Nefrologia*.

[14] Mercadal L., Metzger M., Haymann J. P. (2014). The relation of hepcidin to iron disorders, inflammation and hemoglobin in chronic kidney disease. *PloS One*.

[15] Kell D. B., Pretorius E. (2014). Serum ferritin is an important inflammatory disease marker, as it is mainly a leakage product from damaged cells. *Metallomics: Integrated Biometal Science*.

[16] Gandon Y., Olivié D., Guyader D. (2004). Non-invasive assessment of hepatic iron stores by MRI. *The Lancet*.

